# Long non-coding RNAs: Emerging regulators of diverse programmed cell death pathways in neurons

**DOI:** 10.4103/NRR.NRR-D-25-00233

**Published:** 2025-09-29

**Authors:** Noah C. Mathew, Kevin K. Park

**Affiliations:** 1 Department of Ophthalmology, University of Texas Southwestern Medical Center, Dallas, TX, USA; 2 Department of Neuroscience, University of Texas Southwestern Medical Center, Dallas, TX, USA; 3 Peter O’Donnell Jr. Brain Institute, University of Texas Southwestern Medical Center, Dallas, TX, USA

**Keywords:** apoptosis, lncRNA, necroptosis, neuronal death, non-coding RNA

## Abstract

Long non-coding RNAs have emerged as pivotal regulators of diverse biological processes, particularly in the modulation of regulated neuronal cell death pathways. This review highlights the roles of long non-coding RNAs in programmed neuronal cell death, focusing on apoptosis, necroptosis, ferroptosis, and pyroptosis. Dysregulation of these processes contributes to neurodegenerative disorders and neurological injuries, emphasizing the importance of understanding how long non-coding RNAs influence these pathways. Apoptosis, essential for neuronal development, can lead to pathology when misregulated. Necroptosis, a caspase-independent inflammatory process, involves the modulation of necrosome components. Pyroptosis, mediated by inflammasomes, affects inflammasome assembly and cytokine release. Ferroptosis, driven by iron accumulation and lipid peroxidation, is influenced by changes in antioxidant defenses. By detailing the roles of long non-coding RNAs in these mechanisms, this review underscores their therapeutic potential for mitigating neuronal loss.

## Introduction

Cell death is a fundamental process in the development and maintenance of the nervous system, where a delicate balance between cell survival and death is crucial. The dysregulation of neuronal cell death pathways represents a hallmark of various neurological disorders, often resulting in irreversible loss of neuronal function. Understanding the mechanisms underlying regulated forms of cell death has revealed key mediators critical for cell death progression. These insights are essential for identifying therapeutic targets to mitigate neuronal loss.

**Figure 1:**
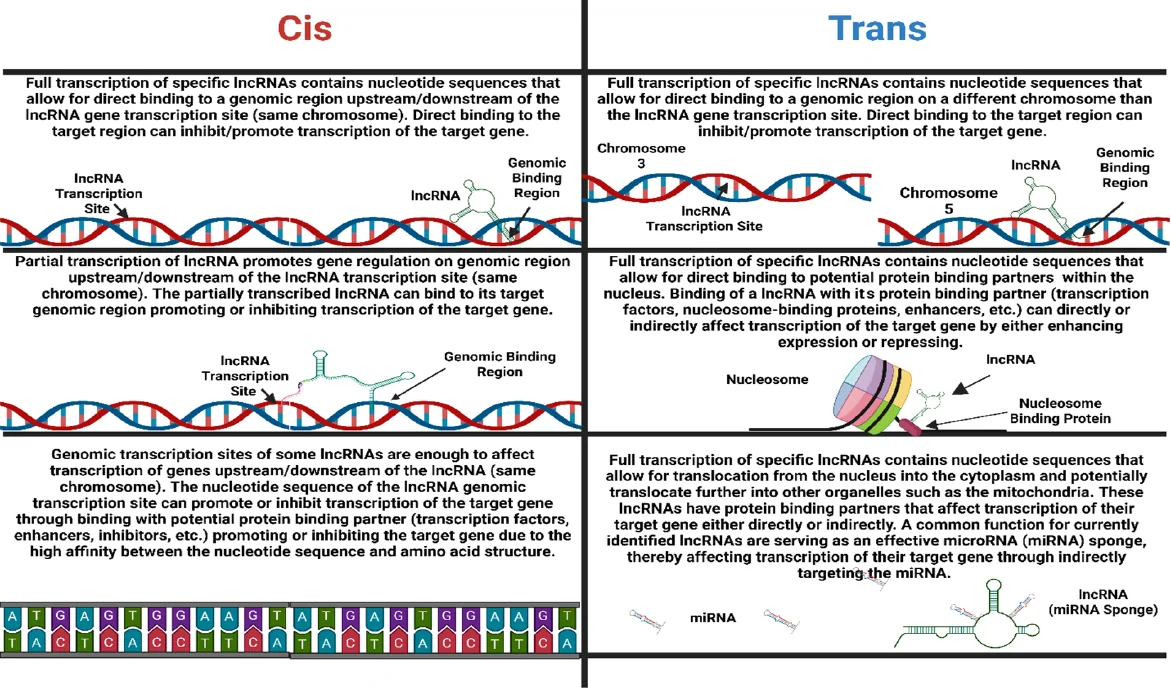
Cis and trans means of gene regulation by lncRNAs. LncRNAs are biologically known for their gene regulatory capabilities. They possess the capability to manipulate gene expression through cis (affecting gene expression on a gene located on the same chromosome as the transcription site of the lncRNA) and trans methods (affecting gene regulation on another chromosome from either direct or indirect methods). Created with BioRender.com. lncRNA: Long non-coding RNA; miRNA: microRNA.

Advancements in high-throughput technologies, such as RNA sequencing and gene expression profiling, have uncovered the importance of non-coding RNA molecules in these processes (Micheel et al., 2021; Gao et al., 2025). Among them, long non-coding RNAs (lncRNAs), which are transcripts exceeding 200 base pairs and lack protein-coding potential, have emerged as significant regulators of cellular processes, including programmed cell death (Mattick et al., 2023). LncRNAs exert their regulatory effects through diverse mechanisms, such as interacting with chromatin, proteins, or other RNA molecules, thereby modulating gene expression at both transcriptional and post-transcriptional levels (Mattick et al., 2023; **Figure 1**).

In recent years, the role of lncRNAs in neuronal cell death has garnered considerable attention. These molecules have been shown to influence various forms of programmed cell death, such as apoptosis, necroptosis, pyroptosis, and ferroptosis. Each of these pathways is characterized by unique molecular mechanisms and outcomes, yet all contribute to the pathology of neurodegenerative diseases and acute injuries. For instance, lncRNAs can function as molecular sponges for microRNAs, scaffold proteins for complex assembly, or regulators of chromatin architecture, thereby impacting cell survival and death (Zhang et al., 2019b; Zuccotti et al., 2025).

This review explores the multifaceted roles of lncRNAs in neuronal cell death, emphasizing their contributions to apoptosis, necroptosis, pyroptosis, and ferroptosis. Furthermore, it highlights the therapeutic potential of targeting lncRNAs to prevent neuronal loss. By understanding the intricate interplay between lncRNAs and cell death pathways, we may uncover novel strategies for treating neurodegenerative diseases and neurological injuries. This growing field of research holds promise for advancing our knowledge of lncRNA biology and its implications in neuronal health and disease.

## Search Strategy

To identify relevant literature for this narrative review, a comprehensive search was conducted using the PubMed database. The search included studies published between the years 2000 and 2025. The following keywords and keyword combinations were used to retrieve relevant articles: Apoptosis and lncRNAs, necroptosis and lncRNAs, pyroptosis and lncRNAs, ferroptosis and lncRNAs, lncRNAs in neuronal cell death, and lncRNAs in neurodegeneration.

## Long Non-Coding RNAs: An Overview

LncRNAs have recently gained prominence as key regulators of gene expression and cellular function (**Figure 1**). Unlike protein-coding RNAs, lncRNAs do not translate into proteins, yet they possess the ability to regulate gene expression through complex interactions with proteins, chromatin, or other nucleic acids. Their unique secondary and tertiary structures, derived from their nucleic acid sequences, facilitate high-affinity binding with target molecules (Mattick et al., 2023).

LncRNAs regulate genes through two primary mechanisms: cis-regulation, where the lncRNA affects gene expression on the same chromosome from which it is transcribed, and trans-regulation, where the lncRNA modulates genes located on different chromosomes. Cis-regulatory actions may involve direct interaction with neighboring genes, binding to intergenic regions to influence upstream or downstream expression, or partial transcription, eliciting effects on nearby genes. In contrast, trans-regulation often includes interactions with chromatin or protein partners, enabling broader influence across the genome. Additionally, some lncRNAs exhibit dual regulatory roles, participating in both cis and trans mechanisms depending on cellular conditions, further enhancing their versatility (Mattick et al., 2023; Zuccotti et al., 2025).

The regulatory functions of lncRNAs extend beyond transcriptional modulation. In the cytoplasm, lncRNAs can function as molecular sponges for microRNAs, sequestering them to prevent their interaction with target mRNAs (Zhang et al., 2019a; Xu et al., 2025). This process allows for the fine-tuning of gene expression and impacts various cellular pathways. For instance, the sequestration of specific microRNAs can lead to enhanced or repressed translation of critical proteins involved in processes such as cell cycle regulation or stress response (Chen and Yan, 2025; Lin et al., 2025; Yang et al., 2025). Additionally, lncRNAs can localize to organelles like mitochondria or the Golgi apparatus, influencing processes such as energy metabolism or protein trafficking (Zhang et al., 2021b; Sun et al., 2022; Wang et al., 2023). This organelle-specific localization enables lncRNAs to play specialized roles, such as modulating mitochondrial biogenesis or maintaining intracellular transport efficiency.

Recent studies have revealed the significant involvement of lncRNAs in regulating neuronal cell death (Sivagurunathan et al., 2024; Baazaoui et al., 2025; Ding et al., 2025). By interacting with key signaling molecules, lncRNAs can either promote or inhibit pathways leading to cell death. Their multifaceted roles extend to forming scaffolds for protein complexes, altering chromatin states, and even participating in feedback loops that amplify or mitigate cell death signals. For example, lncRNAs have been implicated in apoptosis, necroptosis, pyroptosis, and ferroptosis, highlighting their versatile roles in neuronal health and disease. Understanding these mechanisms provides valuable insights into how lncRNAs shape cellular environments and their potential as therapeutic targets for neuroprotection.

## Long Non-Coding RNAs and Apoptosis

Apoptosis is a natural form of programmed cell death that plays a crucial role in neuronal development and general cell turnover. Dysregulation of apoptosis leads to severe anomalies and disorders within the cellular environment. The cellular phenotype of cells undergoing apoptosis includes cellular shrinking, degradation of the nuclear membrane, blebbing of the plasma membrane, chromatin condensation, and the formation of small circular membranes containing organelles and other intracellular components destined for phagocytosis (Reed, 2000).

**Figure 2:**
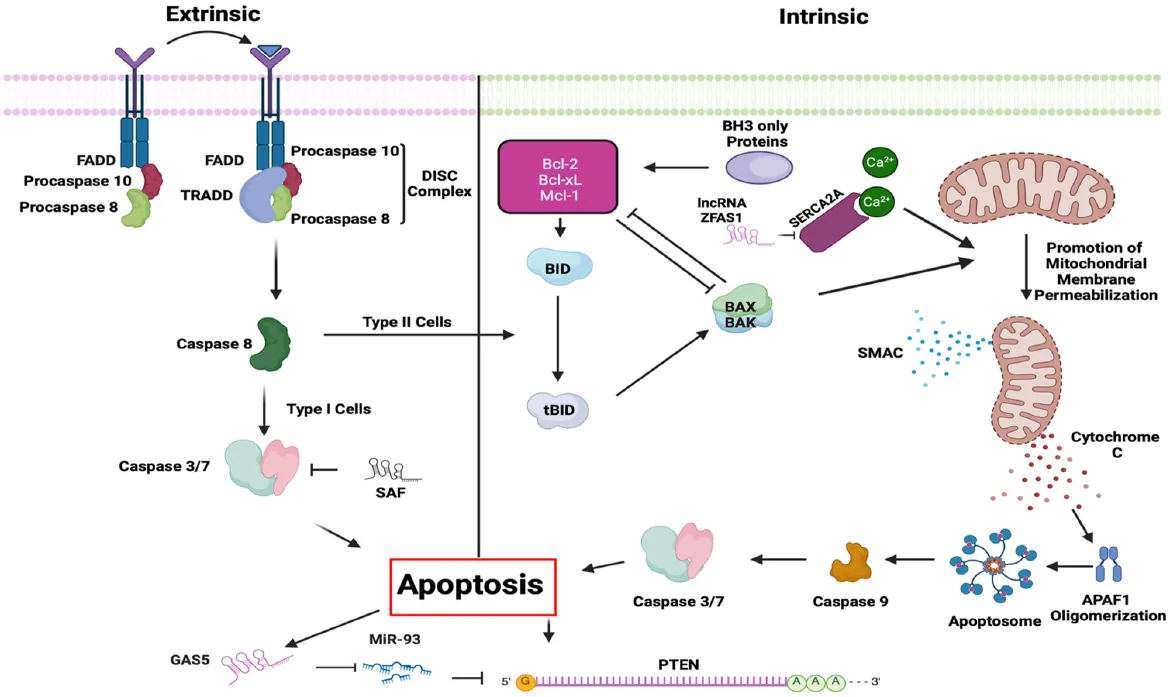
Extrinsic and intrinsic mechanisms underlying apoptosis. Extrinsic means of cellular apoptosis require interaction between a death receptor (e.g., TNFR1 and CD95) and a death ligand (e.g., tumor necrosis factor and CD95L). Binding of the death receptor and ligand allows for the formation of a short-lived complex, the death-inducing signaling complex (DISC). In type I cells, the formation of DISC is enough for the cell to undergo apoptosis through caspase 8, activating caspase 3/7. However, lncRNAs including lncRNA Fas antisense 1 (SAF) has been known to inhibit caspase 3/7 activity leading to cell proliferation (Boliar et al., 2019), In type II cells, active caspase 8 indirectly activates BAX and BAK which then pierce the mitochondrial outer membrane to promote the permeabilization of the mitochondria causing release of SMAC and cytochrome C. Calcium overload within the cell also causes permeabilization of the mitochondria. It has been shown that lncRNA Zinc Finger NFX1-Type Containing 1 antisense RNA 1 (lncRNA ZFAS1) expression inhibits function of calcium chelator sarcoplasmic reticulum Ca^2+^ ATPase 2a (SERCA2a), thereby promoting permeabilization of the mitochondria (Jiao et al., 2019). Release of cytochrome C allows the formation of the apoptosome and eventual activation of caspase 3/7. The intrinsic method of cellular apoptosis is similar to extrinsic type II apoptotic cells. There is no DISC formation; however, intracellular changes cause a rise in expression of BH3 only proteins, which are known to inhibit apoptosis, inhibiting genes. This allows BID to interact with active caspase 8 within the cell, leading to the activation of BAX and BAK, leading to apoptosis. This signaling cascade leads to changes in gene expression that further promotes apoptosis. Two genes that get upregulated are phosphatase and tensin homolog deleted on chromosome 10 (PTEN) and lncRNA GAS5, where inhibition of GAS5 expression leads to an increase in MiR-93 expression, effectively silencing PTEN expression and hindering apoptosis (Cao et al., 2021). Created with BioRender.com. APAF1: Apoptotic protease activating factor 1; BAX: Bcl-2-associated X protein; BAK: Bcl-2 homologous antagonist/killer; Bcl-2: B-cell lymphoma 2; Bcl-xL: B-cell lymphoma-extra large; BH3: Bcl-2 homology 3; BID: BH3 interacting domain death agonist; DISC: death-inducing signaling complex; FADD: Fas-associated protein with death domain; GAS5: growth arrest-specific 5; miR-93: microRNA-93; PTEN: phosphatase and tensin homolog; SAF: scaffold attachment factor; SERCA2A: sarcoplasmic/endoplasmic reticulum calcium ATPase 2A; SMAC: second mitochondria-derived activator of caspases; tBID: truncated-BID; TRADD: tumor necrosis factor receptor 1-associated death domain protein; ZFAS1: zinc finger antisense 1; ZNFX1: zinc finger NFX1-type containing 1.

The mechanisms behind apoptosis have been identified and are categorized into the extrinsic or intrinsic pathways, both utilizing caspase activation to mediate cell death (Jan and Chaudhry, 2019; **Figure 2**). The extrinsic pathway is a receptor-mediated form of apoptosis. Extracellular ligands, referred to as death ligands (e.g., Fas ligand), bind to their respective transmembrane death receptor (e.g., Fas receptor), promoting the binding of cytosolic adaptor proteins, e.g., tumor necrosis factor receptor type 1-associated death domain protein (TRADD), and initiating the generation of the death-inducing signaling complex (DISC). The formation of DISC allows for the activation of procaspase 8 to caspase 8, with the prodomain of procaspase remaining attached to the DISC complex. In type 1 cells, the accumulation of caspase 8 within the cell eventually leads to activation of caspases 3 and 7, culminating in apoptosis. In contrast, in type 2 cells, apoptosis occurs upon initial DISC formation, which leads to the release of pro-apoptotic factors from the mitochondria. In type 2 cells, caspase 8 cleaves BH3 interacting-domain death agonist (Bid), a B-cell lymphoma-2 (Bcl-2) family member, to form a truncated version of Bid (tBid), promoting the mitochondria to release pro-apoptotic factors via the activation of intracellular proteins BCL2-associated X protein (BAX) and Bcl-2-antagonist/killer (BAK) (Elmore, 2007).

The intrinsic pathway of apoptosis occurs in response to intracellular events and changes in the cellular environment (e.g., the presence of BH3-only proteins), which effectively neutralize inhibitors of apoptosis (such as Bcl-2 and Mcl-1; Rasmussen and Gama, 2020). The presence or absence of certain factors (e.g., growth factors, and chemokines) within the cell also promotes the opening of the mitochondrial permeability transition pore, ultimately leading to the release of pro-apoptotic factors, including cytochrome c and second mitochondria-derived activator of caspases (SMAC) (Elmore, 2007). Accumulation of cytochrome c in the cytosol triggers apoptotic protease activating factor-1 oligomerization, apoptosome formation, and subsequent activation of procaspase 9, whereas SMAC accumulation inhibits apoptotic protein inhibitors. Caspase 9 activation directs the cell toward apoptosis through the activation of caspases 3 and 7.

The role of lncRNAs in apoptosis has been observed and extensively reviewed in cancer models, such as the roles lncRNA Fas antisense 1 and lncRNA Zinc Finger NFX1-type containing 1 antisense RNA 1 have in the extrinsic and intrinsic apoptotic pathways, respectively (Boliar et al., 2019; Ghafouri-Fard et al., 2021); however, lncRNAs and their roles in neuronal cell apoptosis are just being discovered (**Figure 2**). Most neurons lack the capacity to be replaced or regenerated once lost, so prevention of neuronal cell loss through apoptosis could mitigate the loss of neuronal function in affected neuronal types (Sirén et al., 2001).

## Long Non-Coding RNA Brain-derived Neurotrophic Factor-Antisense and Apoptosis

Neuronal cell loss in acute spinal cord injury (SCI) causes severe dysfunctions in neuronal activity. In addition to necrotic cell death, delayed neuronal degeneration, commonly occurring around the site of injury, is mediated through apoptosis (Liu et al., 2015b). Recently, PR/SET Domain 5 (PRDM5), a member of the PR domain protein family believed to function as both a tumor suppressor and transcriptional repressor, has been found to be upregulated in various cancers, as well as in the neurons of a rat acute SCI model (Shu, 2015). Silencing PRDM5 effectively reduces neuronal apoptosis and decreases active caspase 3 levels in SCI, suggesting that PRDM5 plays a crucial role in neuronal apoptosis (Liu et al., 2016).

A microRNA (miRNA), miR-130B-5p, has been identified as a negative regulator of PRDM5, thereby alleviating neuronal apoptosis (Zhang et al., 2018b). One of the key functions of lncRNAs is acting as miRNA sponges, which modulate the cellular environment by regulating miRNA availability (Olgun et al., 2018). Brain-derived neurotrophic factor antisense RNA (BDNF-AS) is a lncRNA that has been identified to effectively serve as a miRNA sponge, specifically targeting miR-130B-5p, and functions as a positive regulator of PRDM5 (Zhang et al., 2018b). This study established the presence of a binding domain between miR-130B-5p and BDNF-AS using DIANA Tools software (developed by the Diana Lab at the University of Thessaly) to predict potential miRNA binding targets. This association was further verified through RNA isolation purification (RIP) experimentation involving pheochromocytoma-12 (PC12) cells (Zhang et al., 2018b). Ectopic overexpression of BDNF-AS caused a decrease in miR-130B-5p levels, while the downregulation of BDNF-AS through small interfering RNA (siRNA) treatment resulted in an increase in miR-130B-5p expression.

Given that PRDM5 has already been identified as the target of miR-130B-5p and acts as a pro-apoptotic factor, BDNF-AS is described as a lncRNA that positively regulates PRDM5 function by silencing miR-130B-5p, thereby promoting apoptosis in neuronal cells (Shu, 2015; Liu et al., 2016; Zhang et al., 2018b). These results were further verified by measuring PRDM5 expression, which revealed a positive correlation between BDNF-AS levels and PRDM5 levels. The *in vivo* effects of silencing BDNF-AS in acute SCI rats were also observed. Hind limb locomotor activity improved in rats where BDNF-AS was effectively silenced, indicating the therapeutic potential of targeting BDNF-AS to mitigate neuronal apoptosis in SCI.

## Long Non-Coding RNA DiGeorge Syndrome-associated Noncoding RNA and Apoptosis

Another lncRNA with direct interaction with PRDM5 is DiGeorge Syndrome-Associated Noncoding RNA (DGCR5), a highly expressed lncRNA in the brain with known associations to certain neurological disorders, including Huntington’s disease. In Huntington’s disease, DGCR5 downregulation in specific brain tissues is linked to disease progression (Johnson, 2012; Guida et al., 2017). DGCR5 expression is repressed through the activity of repressor element 1-silencing transcription factor (Johnson, 2012).

To evaluate the role of DGCR5 in acute SCI, expression levels were examined in acute SCI rats. Notably, DGCR5 levels decreased in spinal tissue from acute SCI rats, which was accompanied by increases in PRDM5 and cleaved caspase-3 (c-caspase-3), key markers of apoptotic activity (Crowley and Waterhouse, 2016; Zhang et al., 2018a). To further explore the potential protective role of DGCR5 against apoptosis, experiments were conducted using AGE1.HN and PC12 cells. Cells overexpressing DGCR5 exhibited reduced levels of cell loss compared to control groups (Zhang et al., 2018b).

*In vivo*, intrathecal injection of plasmid cloning DNA to upregulate DGCR5 expression significantly improved Basso-Beattie-Bresnahan scores in acute SCI animals, indicating enhanced motor recovery (Zhang et al., 2018b). Direct interaction between DGCR5 and PRDM5 was confirmed through RNA pull-down assays and RNA immunoprecipitation. Additionally, DGCR5 was found to negatively regulate PRDM5 expression. This was demonstrated via western blot analysis of hypoxia-treated AGE1.HN and PC12 cells, where DGCR5 expression was manipulated using plasmid cloning DNA transfection prior to hypoxia treatment (Zhang et al., 2018b).

These findings suggest that the direct interaction of DGCR5 with PRDM5 prevents apoptosis in neurons following acute SCI. This highlights the critical role of lncRNAs, such as DGCR5, in regulating neuronal apoptosis and their potential therapeutic applications.

## Silencing RNA Growth Arrest–Specific 5 Alleviates Neuronal Cells

One of the mechanisms through which lncRNAs regulate gene expression is by functioning as effective microRNA sponges. One such lncRNA is growth arrest-specific 5 (GAS5), which was first identified in hypoxic and ischemic neonatal rat brain neurons. Silencing GAS5 significantly reduced brain injury and improved recovery of neurological function after injury (Zhang et al., 2019c). This lncRNA is also upregulated in SCI mouse models (Cao et al., 2021).

The binding partner of GAS5 has been identified as microRNA-93. MicroRNAs are another class of non-coding RNA involved in gene expression regulation through post-transcriptional mechanisms, including RNA silencing (Macfarlane and Murphy, 2010). MiR-93 targets and suppresses the expression of phosphatase and tensin homolog (PTEN), a tumor suppressor gene with roles in apoptosis and inflammation. However, GAS5 upregulation silences microRNA-93, thereby promoting PTEN expression (Cao et al., 2021; **Figure 2**).

In both SCI mouse models and hypoxia-treated HT22 cell cultures, targeting GAS5 alleviated inflammation and apoptosis (Cao et al., 2021). Furthermore, targeting GAS5 improved neurological function and provided protection against brain injury in cerebral ischemia/reperfusion mouse models (Zhang et al., 2019c). These findings demonstrate the significant role of GAS5 in regulating neuronal apoptosis and its potential as a therapeutic target across multiple animal models.

## Long Non-Coding RNAs and Necroptosis

**Figure 3:**
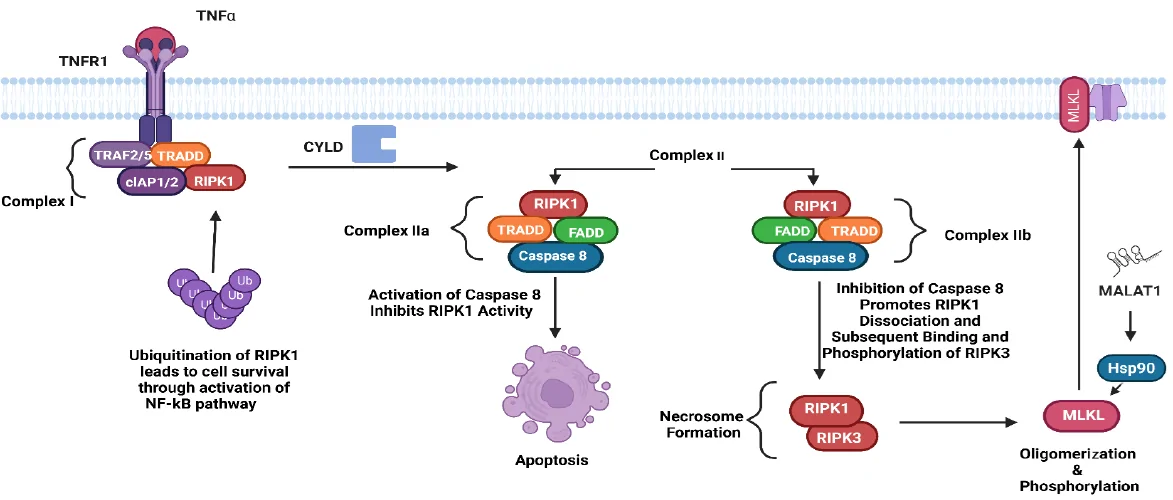
Cellular mechanisms underlying necroptosis. Necroptosis is mediated through the receptor (i.e., TNFR1) and its ligand (i.e., tumor necrosis factor alpha [TNFα]) binding. Binding of the receptor and ligand allows for the formation of a short-lived complex termed Complex I. RIPK1 is one of the proteins in this complex, where ubiquitination of RIPK1 prevents necroptosis through activation of the nuclear factor kappa B (NF-κB) pathway. If cylindromatosis (CYLD) (a deubiquitinase) is expressed in the cell RIPK1 is deubiquitinated, dissociates from Complex I, and is part of another short-lived complex, either Complex IIa or IIb. The difference between the two complexes depends on whether caspase 8 is activated or not. If there is caspase 8 activation, the cell will undergo apoptosis. If caspase 8 is not activated, then RIPK1 dissociates from Complex IIb, binds to, and then phosphorylates RIPK3 (forming another complex called the necrosome). Necrosome formation allows for MKLK oligomerization and phosphorylation. Recent studies have also shown that lncRNA MALAT1 indirectly promotes MLKL oligomerization and translocation through stabilizing HSP90 form and function (Huang et al., 2023). MKLK translocates to the phospholipid bilayer, disrupting membrane integrity. This disruption results in leakage of cellular contents, inflammatory responses, and eventually cell death. Created with BioRender.com. cIAP1/2: Cellular inhibitor of apoptosis proteins 1 and 2; CYLD: cylindromatosis; FADD: Fas-associated protein with death domain; Hsp90: heat shock protein 90; MALAT1: metastasis-associated lung adenocarcinoma transcript 1; MLKL: mixed lineage kinase domain-like protein; NF-κB: nuclear factor kappa-light-chain enhancer of activated B cells; RIPK1/3: receptor-interacting protein kinase 1 and 3; TNFR1: tumor necrosis factor receptor 1; TNFα: tumor necrosis factor alpha; TRADD: TNFR1-associated death domain protein; TRAF2/5: TNF receptor-associated factors 2 and 5.

Necroptosis is a regulated form of necrosis mediated by death receptors on cells or intrinsic changes to the cellular environment that activate specific pathways (Berghe et al., 2014). It is often described as an alternative method of regulated cell death. Necroptosis occurs when the caspase-8-dependent apoptotic pathway is inhibited. Both extrinsic and intrinsic mechanisms of necroptosis rely on interactions between receptor-interacting protein kinase 1 and 3 (RIPK1 and RIPK3) and the activation of mixed lineage kinase domain-like protein (MLKL), which collectively promote necroptosis (Cho et al., 2009; **Figure 3**).

The extrinsic pathway of necroptosis involves ligands, such as Tumor necrosis factor alpha (TNFα), binding to death receptors, e.g., toll-like receptor 4 (TLR4), TLR3, and tumor necrosis factor receptor 1 (TNFR1), forming a short-lived membrane signaling complex (complex I). Complex I includes RIPK1, tumor necrosis factor receptor type 1-associated death domain (TRADD), tumor necrosis factor receptor–associated factor 2/5, and calf intestinal alkaline phosphatase 1/2 (Berghe et al., 2014). Tumor necrosis factor receptor–associated factor 2/5 and calf intestinal alkaline phosphatase 1/2 promote the ubiquitination of RIPK1. If cylindromatosis is absent within the cell, necroptosis is prevented through the activation of the nuclear factor kappa B pathway (Dhuriya and Sharma, 2018). Loss of ubiquitination tags on RIPK1 allows it to dissociate from complex I and form one of two short-lived complexes: complex IIa or IIb. The primary difference between these complexes is the activation (in complex IIa) or inhibition (in complex IIb) of caspase-8. Both complexes include fas-associated death domain, TRADD, RIPK1, and caspase-8 (Dhuriya and Sharma, 2018).

If caspase-8 is present and active, RIPK1 activity is silenced, and the cell undergoes apoptosis instead of necroptosis via caspase-3 activation (O’Donnell et al., 2011). However, if caspase-8 activity is inhibited, RIPK1 dissociates from complex II, binds to RIPK3, and forms the necrosome (Dondelinger et al., 2013). The necrosome complex activates and phosphorylates MLKL, leading to MLKL oligomerization. This allows MLKL to translocate to the plasma membrane, where it forms pores in the lipid membrane and recruits ion channels, causing cellular inflammation (Berghe et al., 2014). In addition to pore formation, the release of inflammatory cytokines further amplifies the inflammatory response in the surrounding cellular environment (Berghe et al., 2014).

The intrinsic cascade of necroptosis is triggered by intracellular stress, such as the accumulation of reactive oxygen species (ROS) (Cai et al., 2014). However, necroptosis is restricted to certain cell types, as it requires the expression of both RIPK1 and MLKL (Berghe et al., 2014).

Recent evidence, primarily from cancer models, highlights the role of lncRNAs as key regulators of necroptosis. As previously mentioned, one mechanism by which lncRNAs regulate gene expression is by acting as effective microRNA sponges. Most lncRNAs involved in necroptosis regulation function by targeting microRNAs through this mechanism (Liu et al., 2015a). Although review articles have documented the roles of lncRNAs in regulating necroptosis in cancer cells (Liu et al., 2015a, 2021), here we focus on their contributions to necroptosis in neurons.

Necroptosis has been shown to cause neuronal cell loss in several neurological conditions, including Alzheimer’s disease (AD) and cerebral ischemia (Balusu et al., 2023; Huang et al., 2023).

## Maternally Expressed Gene 3 and Necroptosis

A recent study demonstrated that the lncRNA maternally expressed gene 3 (MEG3) contributes to necroptosis in neurons afflicted with AD. High-throughput RNA sequencing analysis revealed that MEG3 is upregulated approximately 10-fold in AD mice and 2–3-fold in the temporal gyrus of AD patients (Balusu et al., 2023).

In mature cortical neurons cultured *in vitro*, upregulation of MEG3 resulted in increased cell loss within a few days, along with an upregulation of necroptotic markers, including phosphorylated MLKL (Balusu et al., 2023). Conversely, silencing MEG3 in AD-afflicted neurons alleviated neuronal cell loss and reduced necrosome protein expression. In contrast, MEG3 upregulation promoted both neuronal cell loss and necrosome protein expression. These effects were reversed when the cells were treated with a necrosome protein inhibitor (Balusu et al., 2023).

These findings collectively indicate that MEG3 plays a negative regulatory role in necroptosis. However, further investigation is needed to elucidate the precise mechanisms by which MEG3 influences this process and to develop therapeutic strategies aimed at preventing neuronal cell loss by inhibiting necroptosis.

## Long Non-Coding RNA Mestastasis-associated Lung Adenocarcinoma Transcript 1 and Necroptosis

Metastasis-associated lung adenocarcinoma transcript 1 (MALAT1) is a well-studied lncRNA with known roles in various carcinomas, including lung cancer (Jen et al., 2017). In addition to its involvement in cancer, MALAT1 appears to regulate cell death in neurological conditions, such as cerebral ischemia (Shu et al., 2022).

Genetic silencing of MALAT1 has been shown to reduce cerebral ischemia-reperfusion injury induced by middle cerebral artery occlusion (Wang et al., 2020). Animal studies revealed that silencing MALAT1 in middle cerebral artery occlusion mice reduces cerebral infarction, which is accompanied by decreased phosphorylation levels of RIPK1, RIPK3, and MLKL (Huang et al., 2023). Interestingly, silencing MALAT1 significantly reduced the half-life of heat shock protein 90 (HSP90), suggesting a potential interaction between MALAT1 and HSP90. In the absence of MALAT1, HSP90 becomes destabilized, leading to a lack of RIPK3 activation necessary for necrosome formation (Huang et al., 2023; **Figure 3**).

Another unique role of lncRNAs, including MALAT1, is their ability to stabilize protein complexes. MALAT1 is highly expressed in various cancers and acts as a potent microRNA sponge, co-localizing with nuclear speckles (Zhou et al., 2021). These nuclear speckles are involved in mRNA processing and transcription factor recycling, thereby influencing gene expression within the cell (Spector and Lamond, 2011). RNA-binding domains on nuclear speckles interact with different lncRNAs, eliciting distinct changes in gene expression (Spector and Lamond, 2011; Zhou et al., 2021; Hasenson et al., 2022). However, as with most lncRNAs, the precise mechanisms by which MALAT1 regulates necroptosis remain unclear and require further investigation.

## Long Non-Coding RNAs and Pyroptosis

**Figure 4:**
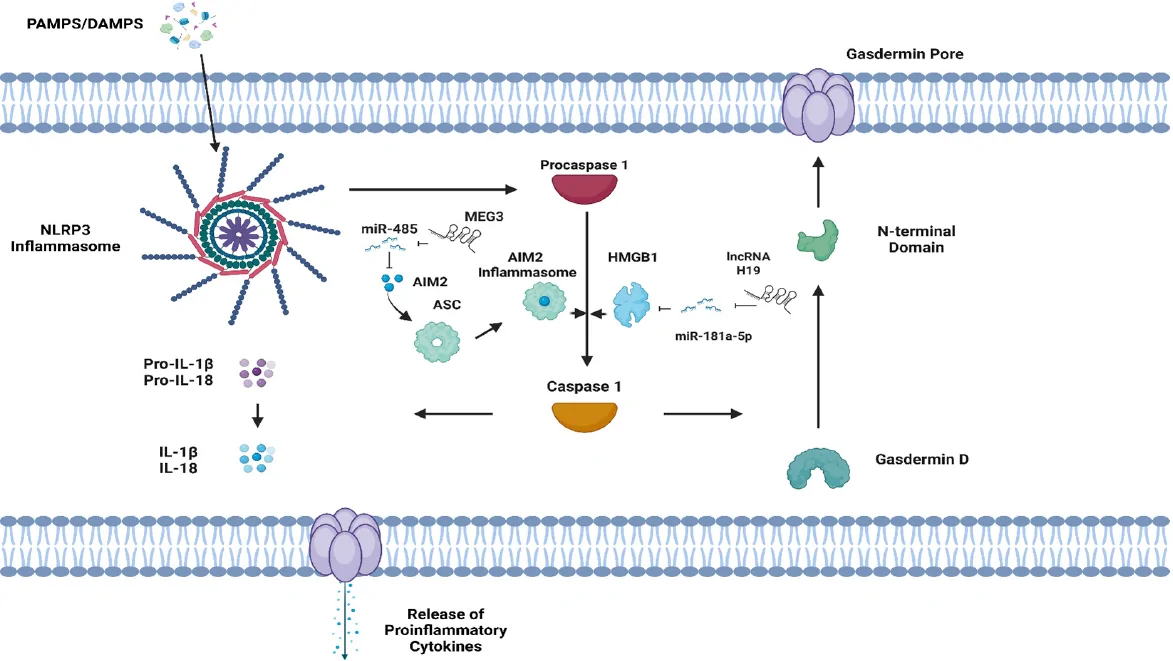
Cellular mechanisms underlying pyroptosis. Pyroptosis is first triggered by the presence of pathogen associated molecular patterns (PAMPS) or damage associated molecular patterns (DAMPS), which allow intracellular mobilization of the inflammasome. Inflammasome activation allows for the dissociation of pro-caspase 1 and eventual activation of caspase 1. Recent research has shown lncRNAs MEG3 and H19 indirectly promote the activation of caspase 1. MEG3 works as a microRNA sponge targeting miR-485, which is known to silence AIM2 expression, thereby preventing AIM2 inflammasome formation, preventing caspase 1 activation (Sagulenko et al., 2013; Liang et al., 2020). H19 also acts as a microRNA sponge targeting miR-181a-5p, which inhibits HMGB1 activity, thereby preventing caspase 1 activation (Hua et al., 2019). Caspase 1 mediates inflammatory cytokine maturation (i.e., pro-IL-1β to IL-1β), as well as asdermin pore formation through interaction with gasdermin D. The N-terminal domain of gasdermin D forms the pore, which then allows the release of proinflammatory cytokines into the extracellular environment. Created with BioRender.com. AIM2: Absent in melanoma 2; ASC: apoptosis-associated speck-like protein containing a CARD; DAMPS: damage-associated molecular patterns; HMGB1: high mobility group box 1; lncRNA H19: long non-coding RNA H19; IL-1β: interleukin-1 beta; MEG3: maternally expressed gene 3; miR-485: microRNA 485; miR-181a-5p: microRNA 181a-5p; NLRP3: NOD-, LRR- and pyrin domain-containing 3; PAMPS: pathogen-associated molecular patterns; Pro-IL-1β: inactive precursor of IL-1β.

Pyroptosis is a form of programmed cell death characterized by a proinflammatory response. It plays a crucial role in the immune system, acting as a defense mechanism against intracellular pathogens. Unlike apoptosis and necrosis, pyroptosis is distinct in its activation of inflammatory cascades and release of proinflammatory cytokines (Bergsbaken et al., 2009; **Figure 4**).

Pyroptosis is primarily initiated by the activation of inflammasomes, multiprotein complexes that function as sensors for pathogen-associated molecular patterns and damage-associated molecular patterns. Extracellular stimulation of toll-like receptors (TLRs) promotes inflammatory cytokine production within host cells (Yu et al., 2021). Similarly, nod-like receptors (NLRs) respond to extracellular signals to stimulate cytokine production (Kufer and Sansonetti, 2007). TLR and NLR stimulation often work in tandem to amplify cytokine production (Kahlenberg et al., 2005; Mariathasan et al., 2006). Importantly, NLR activation also leads to caspase-1–dependent pyroptosis via inflammasome activation (Fink et al., 2008).

The inflammasome Nod-like receptor family pyrin domain-containing 3 (NLRP3) is a pivotal player in pyroptosis. It is composed of pro-caspase-1 and gasdermin D (GSDMD) (Martinon and Tschopp, 2007; He et al., 2015). When activated, the NLRP3 inflammasome triggers caspase-1, a key enzyme exclusive to pyroptosis. A study of caspase-1-deficient mice show no abnormalities in apoptosis or development, highlighting its specific role in pyroptosis (Bergsbaken et al., 2009). Activated caspase-1 cleaves GSDMD, and the N-terminal fragment of GSDMD forms pores in the plasma membrane. This pore formation causes an influx of water and ions, resulting in cell swelling, membrane rupture, and the release of intracellular contents, such as interleukin-1 beta (IL-1β), interleukin-18 (IL-18), and damage-associated molecular patterns (Fink et al., 2008; He et al., 2015). These released cellular contents amplify the immune response, attracting immune cells to the site of infection or tissue damage (Nakanishi et al., 2001).

Recent research has begun to explore the involvement of lncRNAs in regulating pyroptosis, especially in cancer cells (Zhang et al., 2022). One such lncRNA, small nucleolar host gene 7 (SNHG7), is upregulated in hepatocellular carcinoma cells. SNHG7 inhibits NLRP3-dependent pyroptosis by acting as a sponge for microRNA-34a, a known tumor suppressor (Chen et al., 2020). microRNA-34a promotes pyroptosis by silencing the expression of sirtuin 1, a known pyroptosis inhibitor (Chen et al., 2020). By suppressing microRNA-34a, SNHG7 indirectly upregulates sirtuin 1, thereby promoting the proliferation of hepatocellular carcinoma cells (Chen et al., 2020; Zhang et al., 2022).

Another lncRNA, nuclear paraspeckle assembly transcript 1 (NEAT1), has been implicated in pyroptosis. NEAT1 is a nuclear-retained lncRNA known to interact with paraspeckles and influence gene expression. In macrophages, NEAT1 promotes NLRP3 activation by facilitating inflammasome assembly, caspase-1 activation, and inflammatory cytokine production (Clemson et al., 2009).

Studies have also examined the role of lncRNAs in regulating pyroptosis in neurons. Targeting of various lncRNAs has been shown to promote neuroprotection in pyroptosis (Liang et al., 2020; Guo et al., 2022), offering potential therapeutic avenues for mitigating pyroptosis-induced neuronal damage.

## Long Non-Coding RNAs Maternally Expressed Gene 3 and Neuronal Pyroptosis

The role of pyroptosis has been extensively studied in the context of ischemic brain injury. One lncRNA shown to mediate pyroptosis in this condition is MEG3. MEG3 promotes inflammasome activation by functioning as a sponge for microRNA-485 (miR-485) (Liang et al., 2020).

MiR-485 effectively silences absent in melanoma 2 (AIM2) expression by directly binding to the 3′ untranslated region of AIM2, thereby facilitating ischemic tolerance (Dharap and Vemuganti, 2010; Denes et al., 2015). AIM2 is thought to promote pyroptosis through the recruitment of the adaptor protein apoptosis-associated speck-like protein, which contains a domain that activates caspase-1. Several *in vitro* studies have demonstrated that AIM2 expression is silenced by miR-485 activity (Denes et al., 2015; Li et al., 2019; Liang et al., 2020). MEG3 indirectly affects AIM2 expression by sequestering miR-485, preventing it from silencing AIM2 (**Figure 4**). This interaction promotes pyroptosis and inflammation. Silencing MEG3 in human neuroblastoma cell lines (SK-N-SH and SH-SY5Y) enhances miR-485 expression and decreases AIM2 levels, thereby inhibiting pyroptosis and reducing inflammation (Liang et al., 2020). As previously discussed, MEG3 also plays a role in neuronal necroptosis (Balusu et al., 2023), highlighting the multifaceted roles of this lncRNA in regulating different forms of cell death.

## Role of Long Non-Coding RNA H19 in Promoting Pyroptosis

LncRNA H19 has been shown to regulate apoptosis, inflammation, and pyroptosis in neurons following spinal cord injury, as well as in microglia (Wan et al., 2020; Han et al., 2021; Li et al., 2021). Beyond SCI, lncRNA H19 is also associated with glioma, neuroblastoma, and intracranial aneurysms, demonstrating its role in neuronal cell death via manipulation of cell death pathways (Zhong et al., 2021).

After spinal cord ischemia/reperfusion (SCI/R) injury in mice, lncRNA H19 expression increases, while its silencing reduces neuronal pyroptosis *in vitro* (Guo et al., 2022). Mechanistically, H19 promotes pyroptosis by acting as a sponge for microRNA-181a-5p (miR-181a-5p), thereby inhibiting its expression (Guo et al., 2022). MiR-181a-5p plays a neuroprotective role, preventing inflammatory responses. Post-SCI/R injury, miR-181a-5p expression decreases 48 hours after injury, while upregulation of miR-181a-5p targets high-mobility group box 1 (HMGB1), a protein known to activate caspase-1 and facilitate pyroptosis (Hua et al., 2019; Guo et al., 2022; **Figure 4**). Knocking down lncRNA H19 post-SCI/R in mice results in increased miR-181a-5p expression, reduced HMGB1 levels, and improved locomotor activity. This treatment also hinders the spread of neuronal apoptosis (Guo et al., 2022).

Similarly, retinal ischemia/reperfusion (I/R) injury activates the NLRP3 inflammasome and induces pyroptosis in the retina. In these models, lncRNA H19 levels are elevated. Silencing H19 provides neuroprotection to retinal ganglion cells (Wan et al., 2020). H19 promotes pyroptosis in resident retinal microglia, releasing inflammatory cytokines that induce inflammation in neighboring retinal cells via NLRP3 inflammasome activation (Wan et al., 2020).

Mechanistically, lncRNA H19 binds to microRNA-21a-5p (miR-21), suppressing its function and promoting the activation of programmed cell death factor 4 (PDCD4). PDCD4 activation induces the NLRP3 inflammasome via ROS buildup, which leads to mitochondrial membrane collapse in microglial cells (Bergsbaken et al., 2009; Rubartelli, 2012; Wan et al., 2020). Normally, miR-21 inhibits PDCD4 expression, but lncRNA H19 overexpression sponges miR-21, resulting in ROS accumulation, increased PDCD4 expression, and heightened inflammatory cytokine release (e.g., IL-1 and IL-18) (Wan et al., 2020). These events culminate in GSDMD activation and significant cell loss in the ganglion cell layer.

Silencing lncRNA H19 in retinal microglia ameliorates these pathological events, reducing inflammatory cytokines, pyroptosis, and neuronal cell loss (Wan et al., 2020). Collectively, lncRNA H19 is expressed in various tissues under ischemic conditions, and modulating its expression and associated pathways may provide a therapeutic strategy to prevent neuronal cell loss following ischemia.

## Long Non-Coding RNAs and Ferroptosis

**Figure 5:**
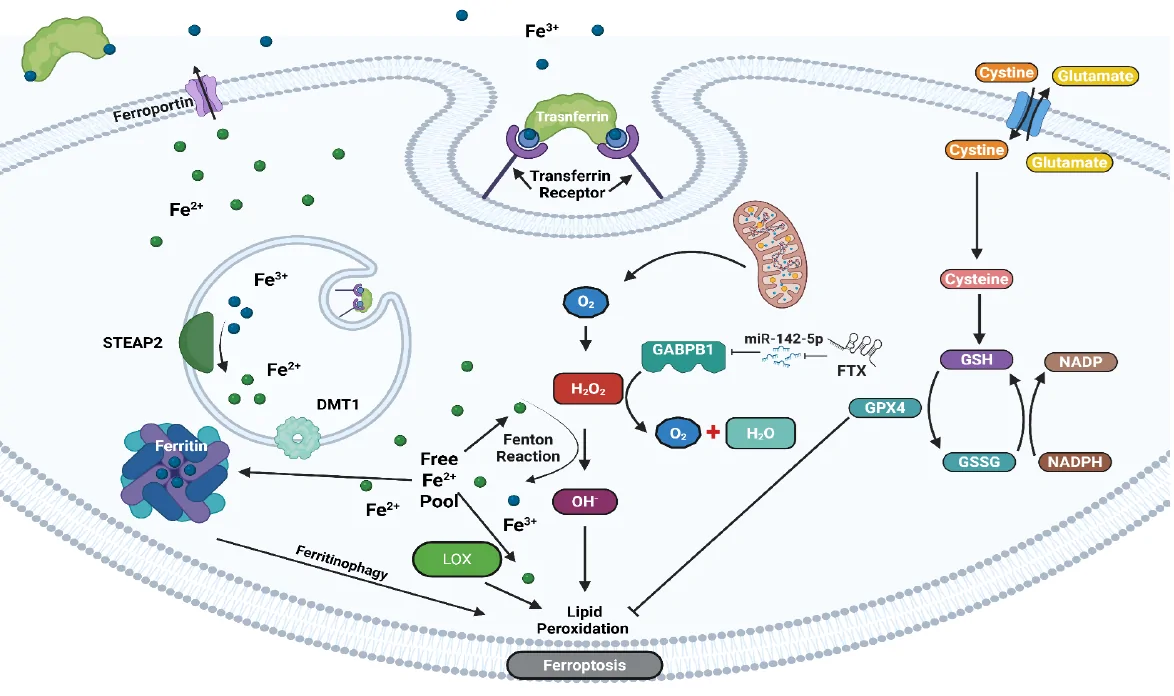
Cellular mechanisms underlying ferroptosis. The cellular phenotype of a cell undergoing ferroptosis includes peroxidation of the cellular membrane, causing a loss of membrane integrity and eventual cellular death. An influx of iron into the cell is a hallmark of ferroptosis. Iron uptake into the cell leads to an accumulation of iron (free Fe^2+^ pool). The accumulation of free iron within the cell triggers several reactions that lead to lipid peroxidation. (1) The free iron is stored in ferritin, a natural protein that stores free iron. Excessive iron stored in ferritin leads to ferritinophagy, which induces lipid peroxidation. (2) The free iron also interacts with lipoxygenases, leading to oxidation of polyunsaturated fatty acids in the bilayer membrane. (3) The free iron can oxidize hydrogen peroxide, causing a buildup of reactive oxygen species. Proteins such as GABPB1 are well known antioxidants that effectively break down hydrogen peroxide into O_2_ and water; miR-142-5p is a known inhibitor of GABPB1 and thus promotes ferroptosis (Qi et al., 2019). Notably, expression of lncRNA FTX works as an microRNA sponge targeting miR-142-5p thus restoring GABPB1 activity (Zhang et al., 2023). However, a buildup of reactive oxygen species leads to lipid peroxidation of the cellular membrane. The accumulation of antioxidants within the cell (via the upregulation of GPX4) counteracts iron accumulation and can prevent the progression of ferroptosis. Created with BioRender.com. DMT1: Divalent metal transporter 1; Fe²⁺: ferrous iron; Fe³⁺: ferric iron; FTX: five prime to Xist; GAPB1: GA-binding protein transcription factor subunit beta 1; GPX4: glutathione peroxidase 4; GSH: glutathione; GSSG: glutathione disulfide; H₂O: water; H₂O₂: hydrogen peroxide; miR-142-5p: microRNA 142-5p; LOX: lipoxygenase; NADP: nicotinamide adenine dinucleotide phosphate; NADPH: nicotinamide adenine dinucleotide phosphate + hydrogen; O₂: oxygen; OH⁻: hydroxide ion; STEAP2: six transmembrane epithelial antigen of prostate 2.

Ferroptosis is a distinct form of programmed cell death, different from apoptosis, and is characterized by iron-dependent accumulation of lipid hydroperoxides. Key processes contributing to ferroptosis include iron metabolism dysregulation, lipid peroxidation, and glutathione depletion (Yan et al., 2021). Ferroptosis is marked by specific cellular morphological changes, such as a round shape before death, shrinking, and increased mitochondrial membrane density, without the structural integrity loss typically seen in apoptotic nuclei (Dixon et al., 2012; **Figure 5**).

While the precise mechanisms driving ferroptosis are still being elucidated, many studies have shown that dysregulation of iron import, storage, and export within the cell plays a critical role. This dysregulation is often due to the increased expression of the transferrin receptor, which enhances iron uptake (Wang et al., 2018; Yan et al., 2021). The buildup of iron inside the cell results in the accumulation of ROS, leading to membrane disruption in both the lipid bilayer of the cell and the mitochondria (Diggle, 2002).

The cell membrane is composed of polyunsaturated fatty acids, and their double bonds make them particularly susceptible to oxidation and ROS-induced damage (Rouzer and Marnett, 2003; Yin et al., 2011). Lipid peroxidation on the cell membrane triggers structural changes such as pore formation, loss of membrane thickness, and increased permeability, all of which lead to regulated cell death (Wong-Ekkabut et al., 2007). Lipid peroxidation occurs when hydrogen atoms are removed from polyunsaturated fatty acids between carbon-carbon double bonds, resulting in phospholipid radicals and lipid ROS buildup (Balihodzic et al., 2022).

Iron accumulation, a hallmark of ferroptosis, promotes lipid peroxidation through mechanisms such as ferritinophagy, interaction with lipoxygenases, and ROS buildup, affecting both the cell and mitochondrial membranes (Wong-Ekkabut et al., 2007; Balihodzic et al., 2022). Glutathione (GPX4) depletion is another key feature of ferroptosis. GPX4 expression has been shown to counteract lipid peroxidation and prevent ferroptosis (Yan et al., 2021). Additionally, proteins such as solute carrier family 7 member 11 (SLC7A11) also help mitigate lipid peroxidation by facilitating GPX4 activity (He et al., 2022).

Several studies have investigated the roles of lncRNAs in regulating ferroptosis, particularly in cancer cells (Mao et al., 2018; Wang et al., 2019; Wu and Liu, 2021; Zhang et al., 2021a; Balihodzic et al., 2022). One example is LINC00336, which has been shown to contribute to ferroptosis in lung cancer. Upregulation of this lncRNA decreases iron concentrations and lipid ROS levels, while increasing mitochondrial membrane potential. It achieves this by indirectly upregulating cystathionine-beta-synthase expression and influencing SLC7A11 expression (Wang et al., 2019).

## Long Non-Coding RNA Five Prime to Xist and Ferroptosis

Only a limited number of studies have explored the role of lncRNAs in promoting ferroptosis in neurons. One such example is lncRNA five prime to Xist (FTX), which has previously been shown to promote ferroptosis in cardiomyocytes and breast cancer cells (Wei et al., 2022; Sun et al., 2023). Since ferroptosis is a characteristic feature of epileptic neurons (Kahn-Kirby et al., 2019), research has focused on examining the involvement of lncRNA FTX in regulating ferroptosis within these cells.

A study by Zhang et al. (2023) revealed that lncRNA FTX is downregulated in epileptic neurons, suggesting that FTX may act to prevent ferroptosis in these cells. *In vitro* experiments with epileptic neurons showed that upregulation of FTX resulted in decreased ROS and iron accumulation, while simultaneously increasing the expression of GPX4 and SLC7A11, both of which inhibit ferroptosis.

It was proposed that FTX functions as a miRNA sponge, targeting microRNA-142-5p (miR-142-5p). Not only is miR-142-5p upregulated in epileptic neurons (Bagla et al., 2018), but FTX was also shown to directly bind to miR-142-5p (Zhang et al., 2023). MiR-142-5p expression silences GA-binding protein transcription factor subunit beta 1 (GABPB1), a known negative regulator of ferroptosis and a crucial factor for neuronal cell proliferation (Liu et al., 2019; Qi et al., 2019; **Figure 5**). The study found a negative correlation between FTX overexpression and miR-142-5p, and a positive correlation with GABPB1 expression (Zhang et al., 2023). These results suggest that FTX plays a neuroprotective role against ferroptosis in neurons.

## Summary and Perspectives

The role of lncRNAs in various cell types undergoing different forms of programmed cell death has gained increasing recognition, particularly in disease and injury contexts where manipulating these lncRNAs can influence cell survival. In the case of neuronal cell loss, multiple lncRNAs have been identified as key drivers of regulated cell death, with the absence of their expression often providing neuroprotection. This relationship highlights the potential therapeutic value of targeting specific lncRNAs to prevent or mitigate neuronal damage.

In apoptosis, lncRNAs such as BDBF-AS, DCGR5, and GAS5 have been shown to promote neuronal cell death, and manipulating their expression can alleviate apoptotic cell loss. Similarly, in necroptosis, the upregulation of MEG3 and MALAT1 contributes to neuronal loss, and silencing these lncRNAs reduces necroptosis. In the case of pyroptosis, the increased expression of lncRNA H19 and MEG3 facilitates pyroptosis progression, while silencing these lncRNAs hinders neuronal death and offers protection. Finally, in ferroptosis, FTX expression inversely correlates with ferroptosis progression, and its downregulation in epileptic neurons has been associated with the promotion of neuronal death through ferroptosis.

Despite the promising neuroprotective effects observed through the manipulation of lncRNAs, identifying which lncRNAs could serve as effective therapeutic targets remains a significant challenge. One strategy for narrowing down potential candidates involves focusing on lncRNAs that are commonly expressed across various diseases or neuronal populations, as these may offer broader therapeutic benefits. In this review, MEG3 and lncRNA H19 were highlighted as lncRNAs with significant roles in multiple forms of regulated cell death, affecting different neuronal populations.

MEG3 has been implicated in promoting neuronal loss through both necroptosis and pyroptosis. Its involvement in multiple forms of regulated cell death suggests it may be an important target for further investigation. Silencing MEG3 has shown neuroprotective effects, making it a promising candidate for therapeutic targeting. The dual role of MEG3 in different forms of cell death also suggests that it could serve as a valuable biomarker for neuronal cell loss, although its complex mechanisms of action pose challenges in fully understanding its roles in these processes.

Similarly, lncRNA H19 was found to be upregulated in various neuronal ischemic injuries that involve pyroptosis. In SCI/R, lncRNA H19 activates HMGB1, which subsequently activates caspase-1, while in retinal I/R, H19 promotes PDCD4 activation and ROS accumulation, both of which trigger inflammasome activation. Although the biological function of lncRNA H19 as a miRNA sponge is consistent across these conditions, the mechanisms behind inflammasome activation differ. This highlights the complexity of lncRNA actions and the challenge in defining their exact mechanisms. However, the widespread expression of lncRNA H19 in ischemic neuronal injuries warrants further exploration of its potential for therapeutic targeting to protect against neuronal damage.

Technological advancements in RNA sequencing and in situ hybridization have made it easier to identify lncRNA expression. However, challenges remain, particularly in identifying lncRNAs that are relevant to human diseases, as evolutionary differences between species may affect lncRNA expression. The detection of many lncRNAs implicated in cell death is further complicated by their low basal expression, which makes it more difficult to identify them in human tissues. Notably, studies have shown that lncRNAs show species-specific variability in both expression patterns and regulatory functions related to cell death mechanisms (Washietl et al., 2014). While some lncRNAs are evolutionarily conserved, many show divergent expression profiles across mammals, with humans expressing certain lncRNAs that are absent or minimally expressed in rodents. These differences may significantly impact apoptotic and autophagic pathways, as species-specific lncRNAs can interact with diverse molecular targets.

LncRNAs likely exhibit variability in their expression patterns and functional impacts on cell death pathways across different strains of the same species and between genders. Strain-specific and gender-dependent differences have been shown to arise from complex genetic backgrounds, hormonal influences, and environmental interactions that collectively shape lncRNA regulation (Issler et al., 2020; Lopez-Royo et al., 2024). In closely related strains, seemingly minor genetic variations can significantly alter lncRNA expression profiles, potentially leading to distinct phenotypic outcomes in apoptosis, necroptosis, or autophagy pathways. Understanding such context-dependent expression and function is crucial for accurately interpreting experimental outcomes and for developing lncRNA-targeted therapies.

LncRNA therapeutics represent an innovative yet nascent field in medical science. Although regulatory bodies have not yet approved any lncRNA-targeted treatments, multiple candidates are advancing through preclinical stages, including disorders of the nervous system. Researchers are investigating various therapeutic strategies such as antisense oligonucleotides, siRNAs, and CRISPR-based technologies to manipulate lncRNA levels or activities (Li et al., 2020; Khaleel et al., 2025). Clinical implementation, however, confronts substantial obstacles, including blood-brain barrier penetration challenges, potential off-target consequences, and gaps in our fundamental understanding of lncRNA mechanisms. While a handful of early clinical studies have begun exploring lncRNAs as potential biomarkers (Anilkumar et al., 2024; Kaur et al., 2025), interventional therapies targeting lncRNAs for CNS conditions have not yet reached clinical trial stages.

In conclusion, while the roles of lncRNAs in regulating neuronal cell death are still being explored, there is immense potential for the development of novel therapeutic approaches. Future research should focus on identifying lncRNAs that are commonly expressed across different diseases and conditions, as well as understanding the mechanisms by which they drive various forms of regulated cell death. By unraveling the complex roles of lncRNAs, particularly those with neuroprotective capabilities, researchers may uncover new therapeutic targets that can prevent or mitigate neuronal cell loss in a variety of neurodegenerative diseases and injuries.

## Data Availability

*Not applicable.*
